# The Impact of Mental Well-Being, Stress, and Coping Strategies on Resilience among Staff Nurses during COVID-19 in Saudi Arabia: A Structural Equational Model

**DOI:** 10.3390/healthcare11030368

**Published:** 2023-01-28

**Authors:** Lailani Sacgaca, Analita Gonzales, Sameer Alkubati, Nojoud Alrashidi, Maha Sanat Alreshidi, Eddieson Pasay-an, Joannes Paulus Hernandez, Mohammad Alboliteeh, Magda Yousif Ramadan, Ameera Khaled Al Onezei, Grace Ann Lim-Lagura, Romeo Mostoles, Reynita Saguban

**Affiliations:** 1 Department of Nursing, Prince Sultan Military College of Health Sciences, Dhahran 31932, Saudi Arabia; 2 Department of Nursing, Faculty of Applied Medical Sciences, University of Tabuk, Tabuk 71491, Saudi Arabia; 3 College of Nursing, University of Hail, Hail 81491, Saudi Arabia; 4 Department of Nursing, Faculty of Medicine and Health Sciences, Hodeida University, Al-Hudaydah P.O. Box 3114, Yemen; 5 Helene Fuld College of Nursing, 24 E. 120th St., New York, NY 10035, USA

**Keywords:** COVID-19, coping strategies, mental well-being, stress, resilience, staff nurses, Saudi Arabia

## Abstract

Previous studies have revealed various factors related to nurses’ resilience and predictors of resilience. However, there are no studies analysing the association of three variables—mental well-being, coping strategies, and stress—with resilience. This study aims to assess the impact of mental well-being, levels of stress, and coping strategies on resilience using path analysis. This study used a cross-sectional approach that involved 763 nurses from 16 major hospitals in the eastern and northern regions of Saudi Arabia during the COVID-19 pandemic. The data gathering was conducted from August to November 2022. The staff nurses possessed positive mental wellness (3.75 ± 1.08), moderate stress levels (3.06 ± 1.21), adequate coping skills (3.33 ± 1.23), and a low level of resilience (2.90 ± 1.040). Age had a small effect on resilience (β = 0.040; *p* < 0.001) but work experience (β = −0.019; *p* > 0.139) and marital status (β = 0.019; *p* > 0.072) were not significant. Conversely, mental well-being (β = 0.043; *p* < 0.001) and stress (β = −0.089; *p* < 0.001) had a small effect on resilience, but coping strategies (β = 0.561; *p* < 0.001) had a large effect on resilience. Therefore, coping strategies must be reinforced at all times to assist nurses and other healthcare professionals in identifying contributing elements that maintain these workers’ resilience in the face of unforeseen and protracted pandemics and other life events.

## 1. Introduction

The largest and most important part of the healthcare delivery system is the nursing profession [1]. Nurses perform multiple responsibilities as managers, counsellors, change agents, and care providers, [2] especially in stressful circumstances. During the COVID-19 pandemic, these roles were greatly threatened, resulting in maladjusted coping strategies and dysfunctional mental and emotional well-being for nurses worldwide [3]. For example, in China, approximately 50% of nurses experienced moderate to high work burnout that resulted in depersonalisation and emotional exhaustion, [4] while in Brazil nearly 50% of nurses experienced burnout and 25% experienced depression [5]. Despite the partial lifting of COVID-19 restrictions, the overall well-being of nurses remains challenged. This is because of the unceasing emergence of highly contagious variants of the virus that are disrupting the mechanisms implemented by governments for the purpose of returning to normal. Accordingly, such challenges are inflicting strain on the global nursing workforce, causing greater occupational risk, professional burnout, low morale, intent to leave the profession, [6] perennial absenteeism, increased probability of medical errors that could compromise patient safety, and low job satisfaction [7]. Therefore, nurses need an optimised level of resilience for adapting to diverse adverse circumstances, decreasing psychological harm, and boosting mental well-being [8].

Coping strategies are either behavioural or cognitive attempts to subjugate and mitigate any potential or actual danger to one’s well-being [9]. The transactional model of stress and coping by [10] identifies challenge and threat as two essential states. These two states emerge from one’s preparedness to respond to actual and potential threats, and from cognitive judgments of the significance of a circumstance. In this model, an individual assesses a situation as benign if achieving a positive outcome requires no action and assesses an event as stressful if achieving a positive outcome requires specific action. Nurses who encountered high levels of stressful experiences had their health negatively affected and exhibited risky behaviours compared to other nurses [11]. High levels of these experiences also impacted their choice to use either adaptive or maladaptive coping strategies to mitigate the consequences of such experiences on their mental well-being [9]. Coping strategies that are approach oriented are prognostic of positive mental well-being, favourable work environments, and excellent safety attitudes [12]. Furthermore, it was revealed that emotion- and problem-focused coping strategies have a full mediating effect on the association between mental well-being and occupation-related stress [13]. Moreover, utilising effective coping strategies is paramount to nurses’ satisfactory work performances, patient and personal safety, and quality healthcare delivery [14].

Resilience as a meta-concept refers to a process, an outcome, and a capability. This study focuses on resilience as a process. As a process, resilience has three phases: before the onset of disaster, during it, and after it. The first phase involves the preparation for a disaster, the second phase involves the response to a situation, and the last phase involves the behavioural, intellectual, managerial, and economic resources for addressing the impact of catastrophe [15], or for coping with and growing after a stressful experience [16]. Numerous studies have shown the relationship of resilience to many variables, such as burnout, job satisfaction, intent to leave the nursing profession, and psychosocial and demographic factors. For instance, studies on the association of job satisfaction, administrative and unit leadership and management support, shared values, and team support with high resilience showed that these factors affected stress in the workplace and intent to stay in the nursing profession [17]. Moreover, studies on psychosocial factors affecting resilience showed that age, level of education, and work experience during the COVID-19 pandemic were positively correlated to resilience [18]. Furthermore, studies on burnout syndrome and resilience in hospital nurses during the COVID-19 pandemic showed that burnout experiences among hospital nurses who cared for COVID-19 patients were high [19]. Multiple other studies have analysed various factors related to resilience and predictors of resilience [20,21,22,23,24]. Gao et al. [25] found that resilience, mental health, and overall well-being were all interrelated. A person’s overall state of well-being can both moderate and mediate the relationship between resilience and mental health. However, to the best of the knowledge of the researchers, there are no studies analysing the impact of mental well-being, stress, and coping strategies on resilience among staff nurses.

The salutogenic theory served as the foundation for this study’s central hypothesis regarding the link between mental well-being, stress, coping strategies, and resilience. This theory emphasizes health promotion, and its central idea centres on stress, which permeates every aspect of human life, yet which many people manage and even thrive on. This served as inspiration for the creation of the salutogenic model, which takes into account the “health ease/disease continuum”. This cyclical process allows people to characterize their current state of health at any point along the continuum, with health-ease at the favourable end and disease at the unfavourable end [26].

According to this concept, the interaction of external risks like stressors, one’s resistance like generalized resistance resources, and the potency of one’s feeling of coherence determine the direction of movement along the continuum. Life experiences lead to the development of generalized resistance resources, which include coping mechanisms expressed in a variety of ways to reduce the negative effects of stressors on well-being. These mechanisms include those that are cognitive, emotional, affective, physical, sociocultural, interpersonal, and relational [27]. A stressful occurrence in the lives of nurses is the COVID-19 pandemic, which could either make or break their coping mechanisms. In this study, it is assumed that mental well-being and stress have an impact on coping strategies, and coping strategies have an impact on resilience.

The purpose of determining the impact of mental well-being, stress, and coping strategies on resilience among staff nurses is to help nurses alleviate the overwhelming negative impact of the current and unforeseen pandemic on the psychological well-being of nurses. An in-depth understanding of the relationship between mental well-being, coping strategies, and stress and the nurturing of resilience can assist healthcare policymakers in prioritising support systems for healthcare workers (especially frontline nurses) and maintaining optimal public health during pandemic and epidemic emergencies. In addition, this study could guide nurses and other healthcare workers in identifying associated factors that could establish and sustain these workers’ resilience in unpredictable and lingering pandemics and other life events. Therefore, this study aims to assess the impact of mental well-being, levels of stress, and coping strategies on resilience using path analysis.

## 2. Methods

### 2.1. Research Design

This study used a cross-sectional approach to explore the impact of mental well-being, stress, and coping strategies on resilience among staff nurses during COVID-19 in Saudi Arabia.

### 2.2. Participants/Setting

Staff nurses working at 16 major hospitals in the eastern (8 hospitals) and northern (8 hospitals) regions of Saudi Arabia served as the participants of the study. A total of 763 nurses participated, as a result of convenience sampling. These nurses were deployed as frontline workers during the initial surge of COVID-19, specifically in emergency departments, medical (COVID) wards, and outpatient departments. Regarding inclusion criteria, nurses were invited if they (a) had been a part of the organisation prior to the initial COVID-19 surge, (b) could comprehend and write English, and (c) were willing to participate.

### 2.3. Data-Gathering Procedure

The researchers used a Google Form survey to gather the data, employing a unique link sent to supervisors. The supervisors of each participating hospital served as the contact persons who helped identify the staff nurses who fulfilled the aforementioned inclusion criteria. Individual responses to the survey were acquired from the participants in their free time. Although permission from the respondents to use their data was not requested, it was assumed that they had given it by filling out and submitting the questionnaire. To increase the response rate, a reminder message was sent through WhatsApp that asked participants to return the completed forms. The participants’ anonymity was maintained with the utmost care. The data gathering was conducted from August to November 2022.

### 2.4. Questionnaires

This research utilised four questionnaires.

#### 2.4.1. Warwick-Edinburgh Mental Wellbeing Scale (WEMWBS) Scoring & Interpretation

The 14-item WEMWBS, which measures the feeling and functional components of mental well-being, is positively worded. There are five response options ranging from ‘none of the time’ to ‘all of the time’ for each of the 14 statements. The WEMWBS is graded by averaging the 14 items’ scores, which range from 1 to 5 for each statement. Total scores range from 14 to 70. Higher scores or means imply better positive mental wellness [28].

#### 2.4.2. Nursing Stress Scale (NSS)

Each of the 34 NSS items is divided into seven categories of work-related stress, which are further grouped into the physical, psychological, and social spheres as follows: (1) “physical environment (workload)”; (2) “psychological environment (death and suffering, inadequate training, lack of support, and uncertainty about treatments)”; and (3) “social environment in the hospital (conflict with physicians and conflict with other nurses)”. The writers discovered only one workload item that was irrelevant: ‘computer breakdown,’ which was replaced by ‘regular job interruptions’ [29]. The responses are based on a Likert scale with four points: never = 0, occasionally = 1, frequently = 2, and very frequently = 3. The total of the scores yields a global index with a range of 0 to 102. A higher score or mean indicates more stressors in the environment.

#### 2.4.3. Coping Strategy Index-Short Form (CSI-SF)

The CSI-SF is designed to follow the original scale’s format, with four four-item subscales: problem-focused engagement (items 1, 2, 8, 9), problem-focused disengagement (items 4, 7, 12, 14), emotional-focused engagement (items 5, 6, 11, 13), and emotion-focused disengagement (items 3, 10, 15, 16). Individuals receive scores for each of the four second-tier subscales (problem-focused engagement, problem-focused disengagement, emotion-focused engagement, and emotion-focused disengagement (range = 4–20)) as well as for each of the two first-tier subscales (engagement and disengagement (range = 8–40)). The four second-tier subscales that were developed each have four components [30]. Better coping skills are indicated by higher means or scores.

#### 2.4.4. Connor-Davidson Resilience Scale (CD-RISC)

The 10 items on the Campbell-Sills and Stein-adapted [31] CD-RISC are on a cumulative 5-point Likert scale, with 0 being almost never and 4 being almost always. The 10-item CD-RISC, employed in an earlier study in the same area, has an excellent overall Cronbach’s alpha of 0.88 in all prior studies conducted in industrialised nations [32]. The respondents provided their responses according to how much they thought each item on the scale applied to them in the month before the survey. Each item received a score ranging from 0 to 40 based on the sum of the responses, with a score of 40 or higher representing the highest level of resilience.

Overall, the four questionnaires had been validated and tested for content and cultural sensitivity. Two experts in nursing education and two from nursing practice were utilized as validators. Accordingly, all four experts unanimously agreed that all the items appeared appropriate for the intended concept. Using 15 staff nurses as a pre-test sample, the instrument’s reliability was assessed, with a Cronbach’s alpha coefficient of 0.94 for WEMWBS, 0.89 for NSS, 0.88 for CSI-SF and 0.90 for CD-RISC.

### 2.5. Ethical Considerations

This research was approved and cleared by the Prince Sultan Medical College of Health Science Institutional Review Board (IRB-2022-NUR–038).

### 2.6. Data Analysis

SPSS version 25 was used to analyse the data. Frequency and percentage were used to treat the descriptive data. Multiple regression was used to test the relationship between each variable. Furthermore, a path model generated through IBM^®^ SPSS^®^ Amos™ 21 was implemented to test the (assumed) relationships of the variables.

**Figure 1:**
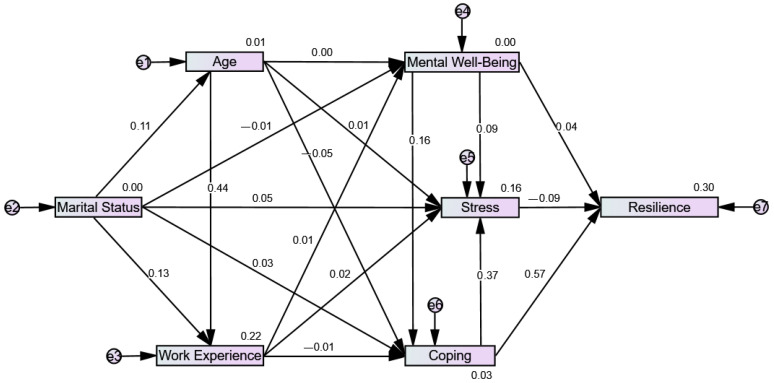
Model on the effects of age, marital status, work experience, mental well-being, stress, and coping on resilience.

A structural equational model generated through IBM^®^ SPSS^®^ Amos™ 21 (i.e., using the dataset file uploaded from IBM^®^ SPSS Statistics 25) was implemented to test the (assumed) relationships of the variables in this study (Figure 1). Correlation results guided the construction of structural equation model. In this model, bootstrapping was implemented using the Monte Carlo approach since analysis involved a data summary for each variable [33]. This was set to run at 2000 samples with a bias-corrected confidence interval at 95%. The cut-offs for interpreting the correlation strength of each variable are according to Mukaka [34].

## 3. Results

Table 1 presents the demographic characteristics of the respondents. Of the 763 respondents, 453 were single while 310 were married. The mean age was 31.77 (SD = 5.88). The largest group of the staff nurses had ≤5 years of work experience (n = 355). Fewer had 5 to 10 years (n = 338) and 11 to 15 years (n = 59) of experience, and the smallest group had 16 to 20 years (n = 11).

Table 2 presents the descriptive results of the measured variables. The staff nurses had positive mental wellness (3.75 ± 1.08), moderate stress level (3.06 ± 1.21), adequate coping skills (3.33 ± 1.23), and a low level of resilience (2.90 ± 1.040).

Figure 1 depicts the causal relations between characteristics of age, marital status, work experience, mental well-being, stress, coping, and resilience. Accordingly, age, marital status, and work experience did not have a direct effect on resilience (as there was no path towards resilience). Moreover, there was an indirect effect of mental well-being on resilience through coping and stress, and an indirect effect of coping on resilience through stress.

## 4. Discussion

This study aims to assess the impact of mental well-being, level of stress, and coping strategies on the resilience of nurses using path analysis. In the present study, staff nurses exhibited positive mental wellness, allowing them to understand what they were going through, which supports findings from previous studies [35,36]. Al Ammari et al. [35] found that nurses experienced a low level of mental stress due to the Saudi government’s immediate response to the pandemic. In general, pandemic healthcare workers were expected to suffer from psychological disorders resulting from other effects of the pandemic including home quarantine, social isolation, altered work schedules, shutdown of public and private organizations, and a focus on hygiene [37]. This is significant because, as healthcare professionals, nurses provide direct care to COVID-19 patients. Therefore, they are more vulnerable when it comes to mental health risks [38].

Although the government quickly established mental health support programmes, the current study result contributes to institutional-level initiatives that should focus on four areas to meet nurses’ growing need for psychological care, education, and therapy. To address nurses’ concerns, the Ministry of Health established a hotline. To meet the growing demand for mental health care, nurses were given access to specialised clinics that focused primarily on preventing burnout or mental breakdown. The support that the Kingdom of Saudi Arabia provided to nurses aligns with some of the strategies proposed in the literature, as it has included the provision of psychological intervention support teams, psychological counselling, a helpline, and online platforms for medical assistance [39]. Nurses were found to have moderate stress, similar to earlier investigations [40,41,42,43,44]. This stress may have stemmed from their concerns about spreading COVID-19 to their loved ones or to other patients. They could have also been stressed about working long hours without appropriate nutrition, facing the deaths of patients and co-workers, experiencing difficulties such as with donning personal protective equipment (PPE) [45], having disagreements over their views with doctors or other nurses, dealing with poor preparation for COVID-19, having a lack of support, or handling a significant workload [46]. Additionally, working in a hospital that accepted COVID-19 patients was significantly associated with increased fear amongst healthcare workers, regardless of whether they interacted directly with these patients [47].

The COVID-19 outbreak, rigorous work, and the vast number of patients caused great physical exhaustion and stress for nurses, which was consistent with other studies on COVID, [48] Ebola [49], and MERS-CoV10 [50]. Furthermore, personnel shortages, time-consuming use of PPE, and extreme perspiration and breathing troubles induced by PPE all contributed to nurses’ stress [51]. This finding contributed to the approach that hospital administrators took when they reported on staff nurses’ stress levels every six months to decide what kinds of interventions to pursue. It is recommended that workshops for nurses be established to help improve their psychological well-being. This study noted better coping skills for nurses, implying that nurses have learned to adapt despite their jobs being affected in new and unexpected ways. Given the fear, frustration, and anger that nurses experience while working in such difficult situations, they are proud of their job and of one another. In keeping with earlier research, the sense of duty engendered by professional ethics during a pandemic [52] improved nurses’ sense of professional identity and pride [53]. Similarly, in comparison with those who expressed less fear and avoided certain issues, those who expressed more fear used active coping techniques [54]. According to Cai et al.’s study, health workers in China considered working during the COVID-19 period to be a moral duty [55]. Therefore, actively teaching the essence of moral duty inspires nurses to realise their own psychological growth may be important in patient care. The pressure of the pandemic may have compelled nurses to use their medical and psychological knowledge to make active or passive psychological modifications.

Nurses used spiritual beliefs, the presence of a support system, and other psychological defences as coping strategies to alleviate stress and negative feelings during the COVID-19 outbreak [56]. Nurses were able to draw from their background knowledge on practices for maintaining good mental health. Since healthcare workers have extensive medical knowledge and a more reasonable and optimistic mindset, nurses were able to adjust their cognitive reasoning in response to the pandemic [57]. Although the participants in this study believed they could handle their emotional stress without professional help, hospitals should monitor their mental health on a regular basis, strengthen support systems for them, and provide them with professional psychological counselling and crisis intervention [55]. To promote emotional release and improve nurses’ mental health, it is best to conduct stress assessments and screenings of nurses immediately after receiving pandemic prevention tasks and to provide professional, flexible, and continuous psychological intervention [48,58,59].

Notably, nurses were found to have a low level of resilience in this study. This might be owing to the fact that the government established mental health support programmes quickly, allowing nurses to be less stressed. Studies have linked lower resilience in nurses to psychological distress like burnout, fatigue, anxiety, and depression [19,60]. The moderate stress levels detected in nurses explain their low level of resilience. In another study, Labrague et al. [61] showed how resilience played a role in lowering nurses’ COVID-19 anxiety levels. In a review study, De Brier et al. [62] determined that, during the previous pandemic (SARS), increasing healthcare professionals’ resilience was essential when it came to assisting them in preserving their mental and psychological health. Prior to the pandemic, nurses already demonstrated resilience; however, the social support they experienced through interactions with colleagues during the pandemic strengthened this resilience even more [52]. During the COVID-19 crisis, this sense of camaraderie and teamwork helped nurses cope with the problems they faced since they cared for each other and shared the load [63]. Consequently, nursing leaders must encourage a sense of belonging and team cohesion among nurses [58]. By assessing nurses’ resilience and offering personalized, effective training and intervention (e.g., increasing resiliency during pandemic) during each session, it is possible to lessen the psychological effects that stressful workplace factors have on nurses while significantly raising their self-efficacy.

Accordingly, age has an indirect effect on resilience, which means that the nurses’ age had a slight impact on their resilience. The finding of this study is similar to Afshari and colleagues [64], denoting that, as nurses mature, both their professional and personal capacities to handle emergency and stressful situations develop. The ability to adapt and respond positively and resiliently in challenging circumstances is greatly aided by the acquisition of such skills [64]. As demonstrated by other studies [65,66], age was also found to be significantly associated with resilience and that trait improved with time in the profession. The development of such abilities facilitates the learning of various coping techniques, which can ease adaptation and give the capacity to function effectively and resiliently under such settings [64]. Hence, it is strongly suggested that younger nurses should be equipped with appropriate training to improve the science and experience in COVID-19 management and coping, thereby enhancing their resilience.

Conversely, work experience and marital status have no direct effects on resilience, which means that the resilience of nurses remains consistent regardless of the nurses’ work experience and marital status. The findings of this study are consistent with the findings of a number of other investigations. In the studies by Aljarboa and associates [32] and Pannell and colleagues [67], the authors arrived at the conclusion that there was no substantial variation in nurses’ level of resilience based on the number of years of experience. In a similar vein, the authors of a number of studies [64,68] suggested that the resilience of nurses remained the same regardless of the nurses’ marital status. This demonstrates that the healthcare system is providing a nurturing and encouraging work environment for registered nurses in order to foster resilience. In doing so, it is recommended that health authorities employ access to training, opportunities for professional progress, a choice of hours, support from peers, feedback on performance, and flexible scheduling for all nurses.

Mental well-being through coping and stress was found to have an indirect effect on resilience, which means that the resilience of the nurses was marginally influenced by mental well-being, coping and stress levels. However, earlier studies [67,69] noted a strong link between the mental health of nurses and their level of resilience. According to the findings of Wu and associates [70], there is a feedback loop that occurs as a result of the influence of resilience on one’s mental health state. One’s mental health status appears to have an effect on resilience, while resilience in turn has an effect on one’s mental health status. Consequently, people who have lower levels of mental health to begin with and who experience adversity later in their lives should receive timely mental health education or intervention in order to improve their level of resilience, as well as their capacity to cope with adversity and their ability to adapt to the environment. Additionally, it was previously discovered that the total resilience score was a strong predictor of the nurses’ reports of feeling stressed [71]. The findings of the present study shed light on the significance of resilience in terms of its role in mitigating the effects of stress on nurses. Therefore, it is essential to evaluate the resilience of nurses in order to determine their mental health.

Coping strategies have an indirect effect on resilience via stress. It is assumed that coping strategies play a fundamental role in nurses’ resilience. However, based on the findings of a number of studies, negative coping mechanisms, such as escaping or avoiding difficult situations or overcommitting oneself to too many activities, are related with poorer mental results [70,72,73]. Positive coping mechanisms, such as a constructive attitude toward the issue at hand, the presence of a social network, the assistance of one’s peers, the ability to collaborate effectively, self-care, problem arbitration, and self-reliance all play a constructive role in the reduction of stress and the enhancement of resilience [73,74,75]. The prior literature has shown that the concepts of resilience and coping methods are intricately connected and have a reciprocal influence on one another [76]. In order to create positive feedback loops, organisations should implement programmes to foster resilience and adaptive coping, such as psychological services, interpersonal intergroup relations, and tailored training.

Overall, this study suggests that healthcare organisations need to help their nurses in coping with the impact of COVID-19. They are in charge of fostering and maintaining worker resilience by offering opportunities for professional growth and motivating strategies that promote a secure and adaptable work environment. When dealing with COVID-19, interventions, such as the creation of workshops, should be implemented to improve medical professionals’ mental health and promote their resilience.

### Study Limitations

The current study only uses self-reported, perception-based data acquired from survey respondents from staff nurses. The results can only be independently validated by the research because no triangulation of findings was used to achieve them. The current conclusions were drawn only from the results of the single survey. In light of the current situation, we advise carrying out a follow-up study to explore the effect of mental well-being, stress, and coping strategies on resilience among staff nurses during COVID-19 utilizing a mixed-methods approach.

## 5. Conclusions

Age has a small effect on resilience but the effects of work experience and marital status were not significant. Conversely, mental well-being and stress have a small effect on resilience but coping strategies have a large effect on resilience. Therefore, coping strategies must be reinforced at all times by nurses’ managers to assist nurses and other healthcare professionals in identifying contributing elements that can help maintain their resilience in the face of unforeseen and protracted pandemics and other life events.

## Figures and Tables

**Table 1:** Demographic characteristics of the staff nurses. n = 763.

	Frequency	Mean	Standard Deviation, SD
Age (Years)	-	31.77 ± 5.88	
Marital Status			
Single	453	-	
Married	310	-	
Work Experience			
1–5 Years	355	6.20 * ± 0.98	
6–10 Years	338		
11–15 Years	59		
16–20 Years	11		

Note: * Grouped data mean estimate.

**Table 2:** Descriptive statistical results for mental wellness, stress, coping strategies, and resilience.

Scale	Mean	SD	Interpretation
Warwick-Edinburgh Mental Well-Being Scale (WEMWBS)	3.75	1.08	Positive mental wellness
Nursing Stress Scale (NSS)	3.06	1.21	Moderate stress
Coping Strategy Index-Short Form (CSI-SF)	3.33	1.23	Adequate coping skills
Connor–Davidson resilience scale	2.90	1.04	Low level of resilience

## Data Availability

The data presented in this study are available on request from the corresponding author.

## References

[1] Farhadi A., Bagherzadeh R., Moradi A., Nemati R., Sadeghmoghadam L. (2021). The relationship between professional self concept and work-related quality of life of nurses working in the wards of patients with COVID-19. BMC Nurs..

[2] Keykaleh M.S., Safarpour H., Yousefian S., Faghisolouk F., Mohammadi E., Ghomian Z. (2018). The relationship between nurse’s job stress and patient safety. Maced. J. Med. Sci..

[3] Bruyneel A., Smith P., Tack J., Pirson M. (2021). Prevalence of burnout risk and factors associated with burnout risk among ICU nurses during the COVID-19 outbreak in French speaking Belgium. Intensive Crit. Care Nurs..

[4] Hu D., Kong Y., Li W., Han Q., Zhang X., Zhu L., Wan S., Liu Z., Shen Q., Yang J. (2020). Frontline nurses’ burnout, anxiety, depression, and fear statuses and their associated factors during the COVID-19 outbreak in Wuhan, China: A large-scale cross-sectional study. EClinicalMedicine.

[5] Dal’Bosco E., Floriano L., Skupien S., Arcaro G., Martins A., Anselmo A. (2020). Mental health of nursing in coping with COVID-19 at a regional university hospital. Rev. Bras. Enferm..

[6] Alameddine M., Clinton M., Bou-Karroum K., Richa N., Doumit M. (2021). Factors Associated with the Resilience of Nurses During the COVID-19 Pandemic. Worldviews Evid. Based Nurs..

[7] Duarte I., Teixeira A., Castro L., Marina S., Ribeiro C., Jácome C., Serrão C. (2020). Burnout among Portuguese healthcare workers during the COVID-19 pandemic. BMC Public Health.

[8] Cooper A.L., Brown J.A., Rees C.S., Leslie G.D. (2020). Nurse resilience: A concept analysis. Int. J. Ment. Health Nurs..

[9] Bérešová A., Kristová J., Mohnyánszki F., Michalková J. (2021). Personal wellbeing and stress coping strategies among nurses working at the departments of anaesthesiology and intensive care. Pielegniarstwo XXI Wieku/Nurs. Century.

[10] Endler N.S., Parker J.D.A. (1994). Assessment of Multidimensional Coping: Task, Emotions, and Avoidance Strategies. Psychol. Assess..

[11] Kowalczuk K., Shpakou A., Hermanowicz J.M., Krajewska-Kułak E., Sobolewski M. (2022). Strategies for Coping with Stress Used by Nurses in Poland and Belarus During the COVID-19 Pandemic. Front. Psychiatry.

[12] Lee T.S.H., Tzeng W.C., Chiang H.H. (2019). Impact of Coping Strategies on Nurses’ Well-Being and Practice. J. Nurs. Scholarsh..

[13] Jang M.H., Gu S.Y., Jeong Y.M. (2019). Role of Coping Styles in the Relationship Between Nurses’ Work Stress and Well-Being Across Career. J. Nurs. Scholarsh..

[14] Puto G., Jurzec M., Leja-Szpak A., Bonior J., Muszalik M., Gniadek A. (2021). Stress and Coping Strategies of Nurses Working with Patients Infected with and Not Infected with SARS-CoV-2 Virus. Int. J. Environ. Res. Public Health.

[15] Duchek S. (2021). Organizational resilience: A capability-based conceptualization. Bus. Res..

[16] Leys C., Arnal C., Wollast R., Rolin H., Kotsou I., Fossion P. (2020). Perspectives on resilience: Personality trait or skill?. Eur. J. Trauma Dissociation.

[17] Gensimore M.M., Maduro R.S., Morgan M.K., McGee G.W., Zimbro K.S. (2020). The effect of nurse practice environment on retention and quality of care via burnout, work characteristics, and resilience: A moderated mediation model. J. Nurs. Adm..

[18] Nourollahi-Darabad M., Afshari D., Chinisaz N. (2021). Psychosocial Factors Associated with Resilience Among Iranian Nurses During COVID-19 Outbreak. Front. Public Health.

[19] Rivas N., López M., Castro M.J., Luis-Vian S., Fernández-Castro M., Cao M.J., García S., Velasco-Gonzalez V., Jiménez J.M. (2021). Analysis of Burnout Syndrome and Resilience in Nurses throughout the COVID-19 Pandemic: A Cross-Sectional Study. Int. J. Environ. Res. Public Health.

[20] Afshari D., Nourollahi-Darabad M., Chinisaz N. (2021). Demographic predictors of resilience among nurses during the COVID-19 pandemic. Work.

[21] Alhawatmeh H., Alsholol R., Dalky H., Al-Ali N., Albataineh R. (2021). Mediating role of resilience on the relationship between stress and quality of life among Jordanian registered nurses during COVID-19 pandemic. Heliyon.

[22] Ang S.Y., Hemsworth D., Uthaman T., Ayre T.C., Mordiffi S.Z., Ang E., Lopez V. (2018). Understanding the influence of resilience on psychological outcomes—Comparing results from acute care nurses in Canada and Singapore. Appl. Nurs. Res..

[23] Atay N., Sahin G., Buzlu S. (2021). The Relationship Between Psychological Resilience and Professional Quality of Life in Nurses. J. Psychosoc. Nurs. Ment. Health Serv..

[24] Delgado C., Roche M., Fethney J., Foster K. (2020). Workplace resilience and emotional labour of Australian mental health nurses: Results of a national survey. Int. J. Ment. Health Nurs..

[25] Gao T., Ding X., Chai J., Zhang Z., Zhang H., Kong Y., Mei S. (2017). The influence of resilience on mental health: The role of general well-being. Int. J. Nurs. Pract..

[26] Antonovsky A. (1979). Health, Stress and Coping.

[27] Antonovsky A. (1987). Unraveling the Mystery of Health—How People Manage Stress and Stay Well.

[28] Tennant R., Hiller L., Fishwick R., Platt S., Joseph S., Weich S., Parkinson J., Secker J., Stewart-Brown S. (2007). The Warwick-Edinburgh mental well-being scale (WEMWBS): Development and UK validation. Health Qual. Life Outcomes.

[29] Escribà V., Más R., Cárdenas M., Pérez S. (1999). Validación de la escala de estresores laborales en personal de enfermería:«the nursing stress scale». Gac. Sanit..

[30] Floyd F.J., Widaman K.F. (1995). Factor analysis in the development and refinement of clinical assessment instruments. Psychol. Assess..

[31] Campbell-Sills L., Stein M.B. (2007). Psychometric analysis and refinement of the connor–Davidson resilience scale (CD-RISC): Validation of a 10-item measure of resilience. J. Trauma. Stress.

[32] Aljarboa B.E., An E.P., Dator W.L.T., Alshammari S.A., Mostoles R., Uy M.M., Alrashidi N., Alreshidi M.S., Mina E., Gonzales A. (2022). Resilience and Emotional Intelligence of Staff Nurses during the COVID-19 Pandemic. Healthcare.

[33] Crowson M. SEM in AMOS when you have Incomplete Data (Video). https://youtu.be/eWHqxrsI0NI.

[34] Mukaka M.M. (2012). Statistics corner: A guide to appropriate use of correlation in medical research. Malawi Med. J..

[35] Al Ammari M., Sultana K., Thomas A., Al Swaidan L., Al Harthi N. (2021). Mental health outcomes amongst health care workers during COVID 19 pandemic in Saudi Arabia. Front. Psychiatry.

[36] Boretti A. (2020). COVID-19 fatality rate for Saudi Arabia. J. Glob. Antimicrob. Resist..

[37] Khanal P., Devkota N., Dahal M., Paudel K., Joshi D. (2020). Mental health impacts among health workers during COVID-19 in a low resource setting: A cross-sectional survey from Nepal. Glob. Health.

[38] Al-Dossary R.N., AlMahmoud S., Banakhar M.A., Alamri M., Albaqawi H., Al Hosis K., Almazan J. (2022). The Relationship between Nurses’ Risk Assessment and Management, Fear Perception, and Mental Well-being during the COVID-19 Pandemic in Saudi Arabia. Front. Public Health.

[39] Shaukat N., Ali D.M., Razzak J. (2020). Physical and mental health impacts of COVID-19 on healthcare workers: A scoping review. Int. J. Emerg. Med..

[40] Almegewly W., Alhejji A., Alotaibi L., Almalki M., Alanezi M., Almotiri A., Albarakah A. (2022). Perceived stress and resilience levels during the COVID-19 pandemic among critical care nurses in Saudi Arabia: A correlational cross-sectional study. PeerJ.

[41] Mohsin S.F., Agwan M.A., Shaikh S., Alsuwaydani Z.A., AlSuwaydani S.A. (2021). COVID-19: Fear and anxiety among healthcare workers in Saudi Arabia. A cross-sectional study. INQUIRY J. Health Care Organ. Provis. Financ..

[42] Rahman M.A., Islam S.M.S., Tungpunkom P., Sultana F., Alif S.M., Banik B., Cross W.M. (2021). COVID-19: Factors associated with psychological distress, fear, and coping strategies among community members across 17 countries. Glob. Health.

[43] Alotni M.A., Elgazzar S.E. (2020). Investigation of burnout, its associated factors and its effect on the quality of life of critical care nurses working in Buraydah Central Hospital at Qassim Region, Saudi Arabia. Open Nurs. J..

[44] Said R.M., El-Shafei D.A. (2021). Occupational stress, job satisfaction, and intent to leave: Nurses working on front lines during COVID-19 pandemic in Zagazig City, Egypt. Environ. Sci. Pollut. Res..

[45] Gilroy R. Nurses on Coronavirus Frontline Facing ‘Abhorrent’abuse from Public 2020. Nursing Times. https://www.nursingtimes.net/news/coronavirus/nurses-fighting-coronavirus-facing-abhorrent-abuse-from-public-20-03-2020.

[46] Magdi H.M. (2022). Stress and Resilient Coping among Nurses: Lessons Learned from the COVID-19 Pandemic. Psych.

[47] Alenazi T.H., BinDhim N.F., Alenazi M.H., Tamim H., Almagrabi R.S., Aljohani S.M., Alqahtani S.A. (2020). Prevalence and predictors of anxiety among healthcare workers in Saudi Arabia during the COVID-19 pandemic. J. Infect. Public Health.

[48] Sun N., Wei L., Shi S., Jiao D., Song R., Ma L., Wang H. (2020). A qualitative study on the psychological experience of caregivers of COVID-19 patients. Am. J. Infect. Control.

[49] Smith M.W., Smith P.W., Kratochvil C.J., Schwedhelm S. (2017). The psychosocial challenges of caring for patients with Ebola virus disease. Health Secur..

[50] Kang H.S., Son Y.D., Chae S.M., Corte C. (2018). Working experiences of nurses during the Middle East respiratory syndrome outbreak. Int. J. Nurs. Pract..

[51] Loibner M., Hagauer S., Schwantzer G., Berghold A., Zatloukal K. (2019). Limiting factors for wearing personal protective equipment (PPE) in a health care environment evaluated in a randomised study. PLoS ONE.

[52] Huang F., Lin M., Sun W., Zhang L., Lu H., Chen W.T. (2021). Resilience of frontline nurses during the COVID pandemic in China: A qualitative study. Nurs. Health Sci..

[53] Peter E., Variath C., Mohammed S., Mitchell M., Killackey T., Maciver J., Chiasson C. (2022). Nurses’ Experiences of their Ethical Responsibilities during Coronavirus Outbreaks: A Scoping Review. Can. J. Nurs. Res..

[54] Moussa M.L., Moussa F.L., Alharbi H.A., Omer T., Khallaf S.A., Al Harbi H.S., Albarqi A.A. (2021). Fear of nurses during COVID-19 pandemic in Saudi Arabia: A cross-sectional assessment. Front. Psychol..

[55] Cai H., Tu B., Ma J., Chen L., Fu L., Jiang Y., Zhuang Q. (2020). Psychological impact and coping strategies of frontline medical staff in Hunan between January and March 2020 during the outbreak of coronavirus disease 2019 (COVID-19) in Hubei, China. Med. Sci. Monit. Int. Med. J. Exp. Clin. Res..

[56] Natividad M.J.B., Aljohani K.A., Roque M.Y., Gamboa H.M. (2021). Feelings, stress, and coping of nurses amidst COVID-19 outbreak in Saudi Arabia. Sudan J. Med. Sci..

[57] Mishra S., Biswas S., Bhatnagar S. (2020). Palliative care delivery in cancer patients in the era of Covid-19 outbreak: Unique needs, barriers, and tools for solutions. Indian J. Palliat. Care.

[58] Liu Q., Luo D., Haase J.E., Guo Q., Wang X.Q., Liu S., Yang B.X. (2020). The experiences of health-care providers during the COVID-19 crisis in China: A qualitative study. Lancet Glob. Health.

[59] Kang L., Li Y., Hu S., Chen M., Yang C., Yang B.X., Liu Z. (2020). The mental health of medical workers in Wuhan, China dealing with the 2019 novel coronavirus. Lancet Psychiatry.

[60] Mealer M., Jones J., Meek P. (2017). Factors affecting resilience and development of posttraumatic stress disorder in critical care nurses. Am. J. Crit. Care.

[61] Labrague L.J., De los Santos J.A.A. (2020). COVID-19 anxiety among front-line nurses: Predictive role of organisational support, personal resilience and social support. J. Nurs. Manag..

[62] De Brier N., Stroobants S., Vandekerckhove P., De Buck E. (2020). Factors affecting mental health of health care workers during coronavirus disease outbreaks (SARS, MERS & COVID-19): A rapid systematic review. PLoS ONE.

[63] Jassar A.S., Perkins K.E., Sundt T.M. (2021). Teamwork in the time of coronavirus: An MGH experience. J. Card. Surg..

[64] Aly H.M., Nemr N.A., Kishk R.M., Bakr Elsaid N.M.A. (2021). Stress, anxiety and depression among healthcare workers facing COVID-19 pandemic in Egypt: A cross-sectional online-based study. BMJ Open.

[65] Hendy A., Abozeid A., Sallam G., Abboud Abdel Fattah H., Ahmed Abdelkader Reshia F. (2021). Predictive factors affecting stress among nurses providing care at COVID-19 isolation hospitals at Egypt. Nurs. Open.

[66] Ali H., Cole A., Ahmed A., Hamasha S., Panos G. (2020). Major stressors and coping strategies of frontline nursing staff during the outbreak of coronavirus disease 2020 (COVID-19) in Alabama. J. Multidiscip. Healthc..

[67] Pannell L.M., Rowe L., Tully S. (2017). Stress Resiliency Practices in Neonatal Nurses. Adv. Neonatal. Care.

[68] Finstad G.L., Giorgi G., Lulli L.G., Pandolfi C., Foti G., León-Perez J.M., Cantero-Sánchez F.J., Mucci N. (2021). Resilience, Coping Strategies and Posttraumatic Growth in the Workplace Following COVID-19: A Narrative Review on the Positive Aspects of Trauma. Int. J. Environ. Res. Public Health..

[69] Ang S.Y., Uthaman T., Ayre T.C., Mordiffi S.Z., Ang E., Lopez V. (2018). Association between demographics and resilience—A cross-sectional study among nurses in Singapore. Int. Nurs. Rev..

[70] Wu Y., Sang Z.Q., Zhang X.C., Margraf J. (2020). The Relationship Between Resilience and Mental Health in Chinese College Students: A Longitudinal Cross-Lagged Analysis. Front. Psychol..

[71] Guo Y.F., Luo Y.H., Lam L., Cross W., Plummer V., Zhang J.P. (2018). Burnout and its association with resilience in nurses: A cross-sectional study. J. Clin. Nurs..

[72] Mirzaei Dahka S., Maroufizadeh S., Pouralizadeh M., Zahedsefat T., Ghanbarpoor Ganjari M., Parsasalkisari E., Ghanbari A. (2022). Mental Health and Resilience among Nurses in the COVID-19 Pandemic: A Web-Based Cross-Sectional Study. Iran J. Psychiatry.

[73] Karabulak H., Kaya F. (2021). The Relationship Between Psychological Resilience and Stress Perception in Nurses in Turkey During the COVID-19 Pandemic. J. Nurs. Res..

[74] Kalaitzaki A., Rovithis M. (2021). Secondary traumatic stress and vicarious posttraumatic growth in healthcare workers during the first COVID-19 lockdown in Greece: The role of resilience and coping strategies. Psychiatriki.

[75] Babore A., Lombardi L., Viceconti M.L., Pignataro S., Marino V., Crudele M., Candelori C., Bramanti S.M., Trumello C. (2020). Psychological effects of the COVID-2019 pandemic: Perceived stress and coping strategies among healthcare professionals. Psychiatry Res..

[76] Vagni M., Maiorano T., Giostra V., Pajardi D. (2020). Coping With COVID-19: Emergency Stress, Secondary Trauma and Self-Efficacy in Healthcare and Emergency Workers in Italy. Front. Psychol..

